# The Profile of Network Spontaneous Activity and Functional Organization Interplay in Hierarchically Connected Modular Neural Networks In Vitro

**DOI:** 10.3390/mi15060732

**Published:** 2024-05-31

**Authors:** Yana Pigareva, Arseniy Gladkov, Vladimir Kolpakov, Victor B. Kazantsev, Irina Mukhina, Alexey Pimashkin

**Affiliations:** 1 Neurotechnology Department, Lobachevsky State University of Nizhny Novgorod, Nizhny Novgorod 603950, Russia; 2 Central Research Laboratory, Cell Technology Department, Privolzhsky Research Medical University, Nizhny Novgorod 603005, Russia

**Keywords:** microfluidics, neuronal tissue engineering, microfluidic cell culture, microchannels, microelectrode arrays, neural network in vitro, modular neural network

## Abstract

Modern microtechnology methods are widely used to create neural networks on a chip with a connection architecture demonstrating properties of modularity and hierarchy similar to brain networks. Such in vitro networks serve as a valuable model for studying the interplay of functional architecture within modules, their activity, and the effectiveness of inter-module interaction. In this study, we use a two-chamber microfluidic platform to investigate functional connectivity and global activity in hierarchically connected modular neural networks. We found that the strength of functional connections within the module and the profile of network spontaneous activity determine the effectiveness of inter-modular interaction and integration activity in the network. The direction of intermodular activity propagation configures the different densities of inhibitory synapses in the network. The developed microfluidic platform holds the potential to explore function-structure relationships and efficient information processing in two- or multilayer neural networks, in both healthy and pathological states.

## 1. Introduction

Understanding brain functions resulting from the interaction of many neuronal elements is a major challenge in neurobiology [1,2,3,4,5]. Despite numerous studies of the complex hierarchical organization of structural and functional brain networks, the relationship between topology and information processing remains poorly understood [6,7,8,9,10,11,12,13]. In recent years, it has been widely shown that brain neural networks have a modular organization, providing low physical connection cost, efficient integration and separation of information [14,15], resistance to damage, and rapid adaptation [16]. In graph theory, modular networks are characterized by densely intra-modular and sparsely inter-modular connections [17,18,19]. Integration refers to the network’s ability to function as a unit and communicate effectively, while segregation refers to its ability to propagate information among localized communities performing specialized tasks.

In vivo studies have shown that the balance of segregation and integration allows for flexible reconfiguration of the functional organization of the brain [20], which is considered necessary for the flexible switch from the resting state to a task-performing state. Thus, the functional organization of the brain in the resting state predicts performance on cognitive tasks [21,22].

The functional connectivity of brain networks is often understood as the set of pairwise statistical dependencies between time series of neurophysiological signals recorded from different regions and can be estimated by simple cross-correlation analysis and more complex measures of directional signal propagation [23]. Methods such as fMRI [24,25,26,27] and EEG/MEG [28,29] are used to study functional connections in brain modular networks. However, access to smaller-scale networks is limited due to a lack of methods for simultaneously registering and identifying dispersed neurons. In vitro methods can be used for more detailed studies of modular network properties. Micro-fabrication techniques allow modeling modular neural networks consistent with several sub-networks connected by a different number of inter-modular connections [30,31,32,33]. Its combination with the multielectrode arrays (MEAs) makes it possible to study functional interaction between modules with high spatial and temporal resolution [34,35,36].

Modular networks in vitro exhibit integration and segregation activity similar to brain networks. This property has been demonstrated by different groups when studying neural networks consisting of two or more modules (up to 60) [32,37,38]. Neural networks in vitro generate spontaneous bursting activity, which can propagate throughout the whole modular network or localize within one module indicating the properties of integration or segregation. The strength of the connection between modules determines the probability of activity propagation between modules. If the connection is too strong, the modular network can degenerate into a homogeneous network, and if the connection is too weak, it can split into separate networks [33]. Another modular network property is high intramodular connectivity compared to connectivity between two modules. Differences in intra- and intermodular interactions are also expressed through delays in the propagation of spontaneous spikes or network bursts [31]. Neurons in the same modules show synchronous activation, while between modules, activity propagates with a time delay on the order of tens of milliseconds. These modularity properties of microfluidic networks in vitro have been widely demonstrated in comparison with homogeneous networks [32,39]. Previous studies have confirmed that the interaction of modules is determined by the number and strength of intermodular connections [31,40,41,42,43]. The interplay of the functional architecture within modules, their activity, and the effectiveness of intermodular interaction has not been widely studied. It has been shown that the activity and functional connections of the modular network change depending on the phenotype of the cells and local activity in the modules [44]. In the multi-module network, the probability of propagation of a burst between modules depends on the intensity of the burst, which is determined through the amplitude of the spikes [32].

Another important aspect of neural networks, in addition to modularity, is the directed propagation and sequential processing of information. Manipulating the shape of microchannels can facilitate the growth of axons from one module (Source) to another (Target) and prevent reverse axonal growth.

In modular networks with undirected connections, the asymmetry of the generation and propagation of spontaneous burst activity was observed, indicating a possible innate property in living neural networks [45,46].

In a multi-module network, some modules were combined into functional subnetworks, influencing the direction of activity propagation between modules. Manipulation with the geometry of microchannels increased the probability of directional activity propagation from the Source to the Target module. Different approaches, such as tapered channels [42], channels with traps for Target axons [47,48], and channels that return Target axons back to the chamber [39,43], have been proposed to improve the efficiency of directional activity propagation in modular networks. How functional connections are formed in modular networks with hierarchical connections and the interaction of modules is still largely unexplored.

In this study, we developed hierarchically connected modular neural networks using a microfluidic platform and explored functional connectivity and integral activity within it.

We found that the intramodular spontaneous activity in such a network can be categorized into two types based on spontaneous burst properties. The type of spontaneous activity appearing in the Source module determines the effectiveness of inter-modular interaction and integration in the network. Hierarchical interactions of modules lead to an uneven distribution of inhibitory synapses in the network. Immunostaining results indicate a decrease in the number of inhibition synapses in the Target module relative to the Source module with an equal ratio of excitatory and inhibitory neurons.

## 2. Materials and Methods

### 2.1. Microfluidic Device Fabrication

The microfluidic chips were fabricated via polydimethylsiloxane (PDMS) molding techniques. Standard two-layer lithography was used for mold fabrication [49]. The microfluidic chip consisted of two chambers (220 μm height) for neuronal cell culturing and sixteen microchannels (5.5 μm height) for neurites. According to the predefined direction of axon growth, the modules were labeled Source and Target modules. We previously showed that the microchannels with asymmetric shapes provide unidirectional axon growth between neurons [50]. Here, we used the same type of microchannels that consisted of three segments: two segments had a narrow triangular shape (200 μm length, 40 μm width) and one segment had a large triangular shape (200 μm length, 150 μm width). The large segment had two traps to capture the axons of neurons.

PDMS (Sylgard 184, Dow Corning, Midland, MI, USA) Base and Curing agent were mixed (10:1) and cured on a master mold at 80 °C for 2 h. After peeling, PDMS was kept in an oven at 100 °C for 12 h to finalize the cross-linking of uncured oligomers [51].

**Figure 1:**
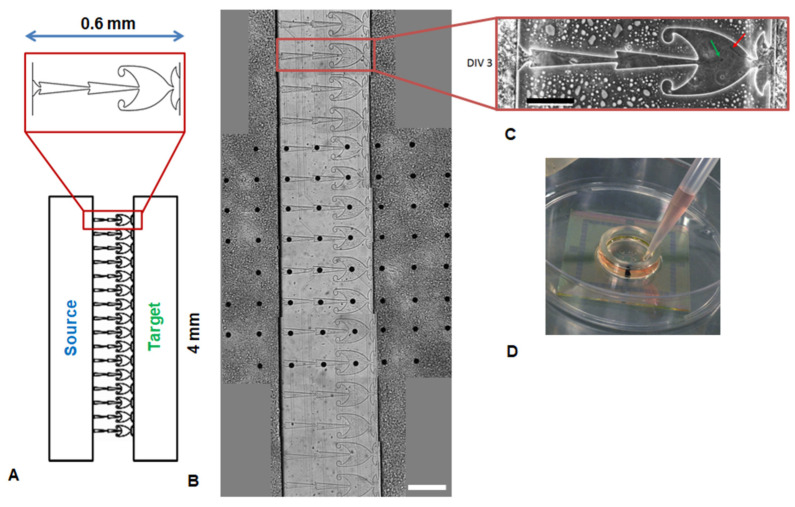
(**A**) Scheme of the microfluidic chip. Asymmetric microchannels connect the Source and the Target chambers. (**B**) Microphotograph of modular neural network in the microfluidic chip mounted on MEA at 10 DIV (scale bar 200 μm). (**C**) Neurites grow through the microfluidic channel. The axons from the Source chamber (green arrow) grow fast alight triangular sections. The neurites from the Target chamber (red arrow) are mostly left in the big section (scale bar 100 μm). (**D**) Photograph of a microfluidic chip mounted to the MEA.

The wells for cell seeding were cut with a puncher (2 mm in diameter) at the two opposite corners of each chamber. The chips were cleaned with compressed air (2 kPa, filters 1 and 0.05 μm) and 3M Scotch (3M, Maplewood, MN, USA). We mounted the PDMS chips with microelectrode arrays (MEAs) to study the electrical activity of the cultures. The chips were manually aligned with the MEAs (Figure 1D), which were composed of 60 electrodes (TiN electrodes, diameter 30 µm with 200 µm in-between, Multichannel Systems, Kusterdingen, Germany) under a binocular so that each of 16 microchannels encompassed 3 electrodes. A drop of 96% ethanol was applied before the alignment.

The surfaces of the prepared PDMS devices were coated with the adhesion-promoting molecule polyethyleneimine (PEI, 1 mg/mL, Sigma-Aldrich, P3143, St. Louis, MO, USA) at 4 °C overnight. The PEI was washed three times with deionized water. Subsequently, the chips were filled with laminin (20 μg/mL, Sigma-Aldrich, L2020, USA) and incubated at 37 °C for 30 min.

### 2.2. Cell Culturing

Hippocampal cells were dissociated from embryonic C57BL/6 mice (E18) of unknown sex and plated in the chambers of PDMS chips at an initial density of 7000–9000 cells/${mm}^{2}$. Experiments were performed following the protocol approved by the Bioethics Committee of the National Research Lobachevsky State University of Nizhny Novgorod (Russia). For procedure details of culturing, see [52]. The cells were seeded in Neurobasal medium enriched with 2% B-27 (Invitrogen, A3653401, Waltham, MA, USA), 1% glutamine (Invitrogen, 25030-024, USA), 5% fetal calf serum (Invitrogen, A3160801, USA), and gentamicin 20 μg/mL (AppliChem, A1492, Darmstadt, Germany). On the next day, half of the medium was replaced with a new one in which the cells were cultured. The cells were cultured under constant conditions of 37 °C, 5% CO_2_, in a cell culture incubator (3552-2, SHEL LAB, Cornelius, OR, USA). MEAs were constantly covered with PDMS lids to attenuate osmotic drift in long-term cell cultures [53]. The cells were cultured in Neurobasal medium enriched with 2% B-27 (Invitrogen, A3653401, USA), 1% glutamine (Invitrogen, 25030-024, USA), 0.4% fetal calf serum (Invitrogen, A3160801, USA), and gentamicin 20 μg/mL (AppliChem, A1492, Germany). Half of the culture medium was replaced twice a week.

Phase contrast images were obtained from neurons growing on coverslips using a Cell IQ system (ChipMan Technologies, Tampere, Finland) with ×20 objective (Nikon CFI Plan Fluorescence ELWD ADL).

### 2.3. Immunohistochemistry

For immunostaining, we used cultures grown in microfluidic chips bonded to the glass coverslips coated by PEI. Then, the chips were mounted via a reversible method to remove the chips from coverslips to access the neurites in the microchannels for staining. On 21 days in vitro (DIV), the neuronal cultures were taken for immunostaining. The microfluidic chip was removed to access the neurites in the microchannels for immunohistochemistry (ICC) staining. The culture medium was first washed with warm (37 °C) PBS three times. Then, cells were fixed with warm (37 °C), freshly prepared 4% paraformaldehyde (141451.1211, AppliChem, Germany) for 15 min at room temperature, and then washed with PBS three times for 5 min. Cells were permeabilized with 0.1% Triton (X-100, Sigma-Aldrich, Darmstadt, Germany) in PBS with 2% BSA (Bovine Serum Albumin) for 20 min and then washed with PBS three times for 5 min.

To assess excitatory and inhibitory synapses in mature neuronal culture, the primary antibodies were anti-vesicular glutamate transporter 1 (VGLUT1) rabbit IgG (Abcam, ab180188, Cambridge, UK), anti-vesicular GABA (Gamma-Aminobutyric Acid) transporter (VGAT) mouse IgG (Abcam, ab211534, UK), and anti-microtubule associated protein 2 (MAP2) chicken IgG (Abcam, ab5392, UK), respectively. The secondary antibodies were goat Alexa Fluor 488-labeled anti-rabbit IgG (Abcam, ab150077, UK), goat Alexa Fluor 647-labeled anti-mouse IgG (Abcam, ab150115, UK), and goat Alexa Fluor 555-labeled anti-chicken (Abcam, ab150170, UK) for VGLUT1, VGAT, and MAP2, respectively.

To assess excitatory and inhibitory neurons in mature neuronal culture, the primary antibodies were anti-Ca2+/calmodulin-dependent protein kinase II transporter (CaMKII) rabbit IgG, (Abcam, ab134041, UK), and anti-neuronal nuclear antigen (NeuN) rat IgG2a (Abcam, ab279297, UK), respectively. The secondary antibodies were goat Alexa Fluor 647-labeled anti-rabbit IgG (Abcam, ab150083, UK) and goat Alexa Fluor 647-labeled anti-rat IgG (Abcam, ab150166, UK) for CaMKII and NeuN, respectively.

Cells were incubated with primary antibodies at room temperature for 2 h and then washed with PBS buffer three times for 5 min. Cells were incubated in the dark with secondary antibodies at room temperature for 1 h and then washed with PBS twice and with deionized water to remove salt. After that, the cells were fixed in a mounting medium with DAPI (4′,6-diamidino-2-phenylindole, Abcam, ab104139, UK).

Fluorescence images of presynaptic terminals were acquired using a confocal laser-scanning microscope (Zeiss LSM 800, Jena, Germany) through Alpha Plan-Apochromat 63×/1.46 Oil Corr M27 objective. The image size was 1024 × 1024 pixels of 101.41 × 101.41 μm fields of view. Images were taken with a 3.15 µm pinhole. Z-stacks reached 13–15 slices (2.04–2.38 µm).

To assess excitatory and inhibitory synapses we manually counted (1) the number of VGLUT1 puncta per μm MAP2-immunopositive dendrite length; (2) the number of VGAT puncta per μm MAP2-immunopositive dendrite length. To count VGLUT1 and VGAT punctas, we selected 5 sections of dendrites (60–90 µm, 44 dendrite sections in total) in the Source and Target networks of 7 cultures.

To assess excitatory and inhibitory neurons, we manually counted the number of cells that expressed CaMKII and NeuN in the region of interest with an area of 0.01 ${mm}^{2}$ (6 cultures).

### 2.4. Electrophysiology

Spiking activity (10 min) was recorded on 15, 20, and 25 DIV from 59 (1 reference) TiN electrodes of the MEA system (Multichannel Systems, Kusterdingen, Germany) at a sample rate of 20 kHz. The electrodes were 30 µm in diameter, and the distance between the electrodes was 200 µm. Recordings were performed outside the incubator. MEAs were covered with PDMS lids to prevent evaporation of the medium. The bottom of the MEAs was heated to 37 °C during the recording with a commercially available heating module (TC02, Multichannel Systems, Germany). An air mixture with 5% CO_2_ was continuously supplied under the aluminum covers. The recordings were started at least 15 min after placing the cultures into the MEA system to exclude the mechanical impact on the activity.

### 2.5. Analysis of Spiking Activity

Detection of the recorded spikes was based on the threshold calculation of the signal median. Details of the spike and the burst detection method were described in our previous study [52]. The firing rate was calculated as the number of spikes per second. The spontaneous bursting activity was characterized as a number of spikes per burst in each burst separately for the Source and the Target module. The total number of spikes was normalized to the number of electrodes in the module (SBE). Burst duration (BD) was calculated as an interval from the beginning to the end of each detected burst [52], and burst per minute was calculated as the number of detected bursts divided by recording time.

All signal analysis and statistics were performed with custom-made software in Matlab v. 8.2 (R). The probability of the burst propagation was estimated by previously proposed methods [47]. The propagation probability (PP) of the burst in a Source–Target direction and Target–Source direction was estimated as the number of bursts propagated from the Source to the Target divided by the burst number in the Source (PPST) and the bursts propagated from the Target to the Source divided by the burst number in the Target (PPTS), respectively. We found bursts propagated between modules in 7 out of 10 cultures.

### 2.6. Cross-Correlation Analysis

Conditional firing probabilities (CFP) were used to determine functional connectivity in each of the 10 min recordings of spontaneous activity [54]. CFPs were calculated for all possible pairs of electrodes as the probability of recording a spike at electrode j at t = τ (τ > 0), given that one was recorded at electrode i at t = 0. Two neurons were defined as functionally connected if a CFP curve was not flat. Functional connections were characterized by strength and latency.

CFPs were calculated in each module separately and between modules. The Source–Target connections were evaluated as follows: all electrodes in the Source module have been adopted as i and all electrodes in the Target module have been adopted as j. Due to the negligible number of bursts propagating from the Target to the Source we did not analyze backward connections (Target–Source).

### 2.7. Burst Cluster Analysis

In each record of spontaneous activity, one or two burst types were identified using the K-means clustering algorithm according to the number of spikes in bursts. Cluster separation was carried out by calculating the Davies–Bouldin index. The DB index evaluates the relationship between the internal distance within clusters and the distance between clusters. Exceeding the value of 0.5 by the index determined the presence of one cluster in spontaneous activity. If the index was in the range from 0 to 0.5 and the percentage of bursts in each of the clusters was at least 10% of the total, it was considered that the activity included two clusters.

### 2.8. Statistical Analysis

In the Results section, data are expressed as medians. Statistical analyses were performed with custom-made software in Matlab v. 8.2 (R). The significance level was set at *p* < 0.05. Statistics were performed by medians of a Mann–Whitney test.

## 3. Results

### 3.1. Differences in Synaptic Structure of the Source and Target Modules

We investigated modular hippocampal networks grown on the microfluidic chip mounted with the MEA. Neural subnetworks (modules) were isolated from each other in the chambers and connected by neurites grown through 16 microchannels (Figure 1A,B). The specific asymmetrical shape of the microchannels facilitated axonal growth from the Source to the Target module and prevented neurite growth from the Target module beyond the wide section of the microchannel (Figure 1C). As we showed earlier, the dendrites from the Source module did not spread further than the middle of the microchannel [55].

**Figure 2:**
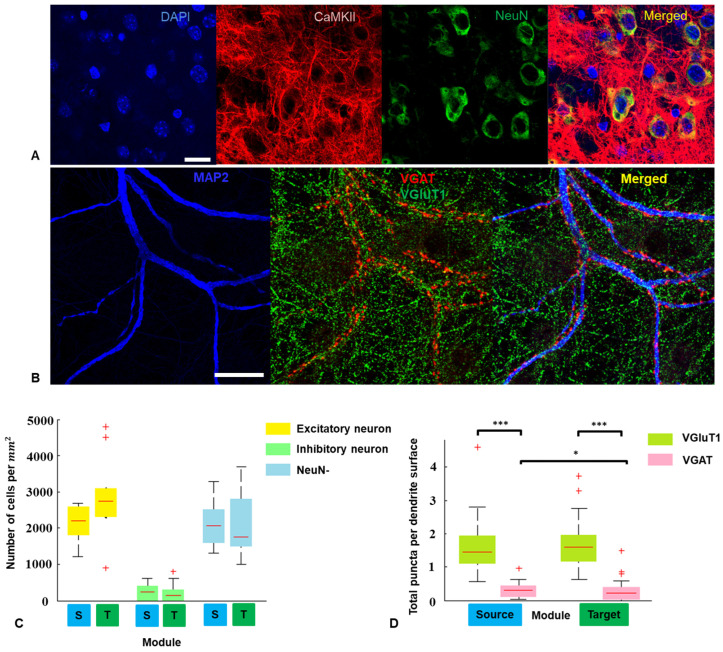
(**A**) Microscopic images visualizing the expression of nuclear marker DAPI (blue), CaMKII (red), and neuronal marker NeuN (green) in the Source module of the neuronal network in vitro at 21 DIV. Scale bar = 20 μm. (**B**) Immunocytochemistry GABAergic neurons marker VGAT (red) and glutamatergic neuronal marker VGluT1 (**C**) along with MAP2 neuronal cytoskeletal marker (blue) in the Source module at 21 DIV. Scale bar = 20 μm. (**C**) Number of excitatory neurons, inhibitory neurons, and NeuN-cells in the Source and the Target modules per ${mm}^{2}$ region of interest at 21 DIV. (**D**) Total VGluT1 and VGAT puncta per μ$m^{2}$ on dendrites in the Source and the Target modules at 21 DIV (n = 7 networks, 82 dendrites, Mann–Whitney test, *—*p* < 0.05, ***—*p* < 0.001). On each box, the central red line indicates the median, the ‘+’ symbol indicates the outliers.

Next, we estimated the number of neurons in connected subnetworks, as well as the number of excitatory and inhibitory synapses, to determine whether the activity of the Source module changed the structure of the Target module. To determine the number of excitatory and inhibitory neurons in the modules, we assessed the expression of calcium–calmodulin-dependent protein kinase II (CaMKII) and the neuronal differentiation marker NeuN in cultures on 21 DIV (Figure 2A). The expression of CaMKII in NeuN-positive cells indicated the phenotype of excitatory neurons, and the absence of expression indicated the phenotype of inhibitory neurons. NeuN-negative cells were defined as glial. We found that the number of excitatory neurons, inhibitory neurons, and glial cells were not statistically different in both modules (Figure 2C; n = 5 networks, Mann–Whitney test).

Next, the densities of VGluT1- (excitatory) and VGAT- (inhibitory) immunopositive puncta on dendrites at 21 DIV were analyzed. An example of a fluorescent image of dendrites and presynaptic terminals is shown in Figure 2B. We observed a significantly higher density of VGAT puncta on dendrites in the Source module than in the Target module (Figure 2D; n = 7 networks, 82 dendrites, Mann–Whitney test, *p* < 0.05). At the same time, the VGluT1 puncta in the modules were not different (Mann–Whitney test, *p* = 0.6886).

### 3.2. Two Types of Spontaneous Bursting Activity

**Figure 3:**
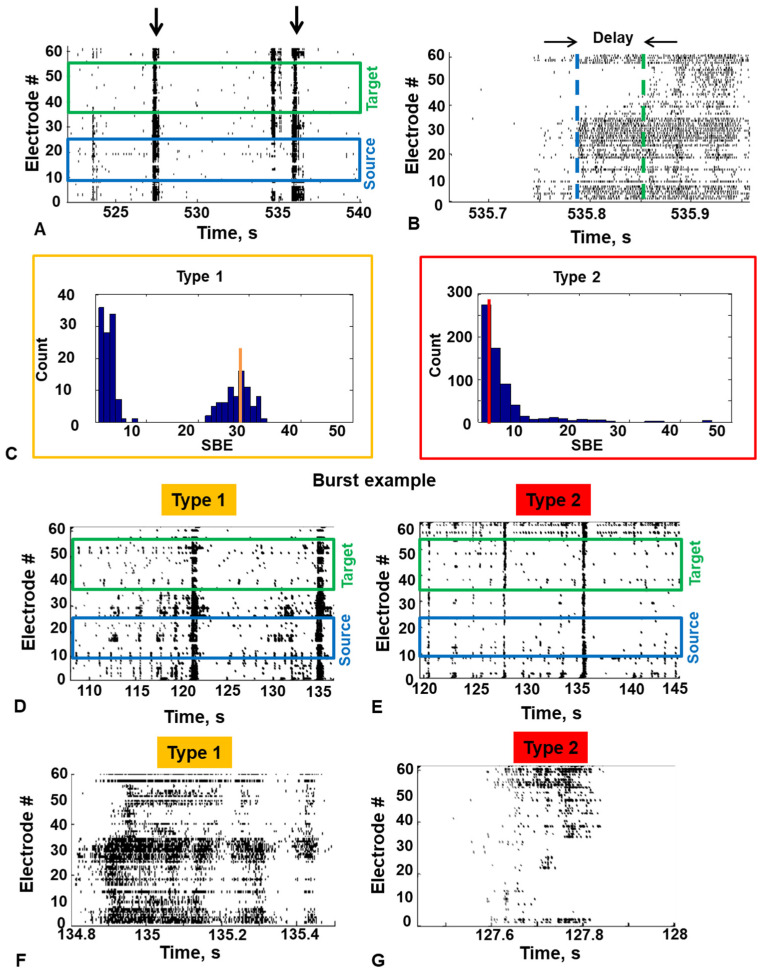
Bioelectrical activity in modular networks. (**A**) The raster of spiking activity in the modular network. Integrated burst propagated through both the Source and the Target modules marked by arrows. (**B**) The delay of burst propagation from the Source (blue line) to the Target module (green line). (**C**) Examples of the distributions of the SBE of the type 1 (yellow) and the type 2 (red) of activity. The vertical line denotes the median of the SBE distribution (large burst for type 1 and all burst for type 2). (**D**–**G**) The raster of spiking activity and an example of a single burst of type 1 and type 2.

Then, we evaluated the formation of functional connections between the modules. The spontaneous electrophysiological activity was recorded on 20 and 25 DIVs using the MEA system. An example of the bursting activity recorded by MEA on 20 DIV is shown in Figure 3A,B. Part of the bursts propagated within just one module (intramodular activity), but some involved both modules (intermodular activity) (Figure 3A, marked with arrows). The coexistence of both intra- and inter-modular activity in most of our networks reflected the main property of modular systems. Inter-modular activity was not observed in 3 out of 10 cultures, so they were excluded from further consideration. To investigate how the characteristics of activity can determine intermodular interaction, we separated intramodular activity recorded on 20 and 25 DIVs into two categories based on bursting activity characteristics. The Source and Target activity were analyzed separately. Using the K-means clustering method, we found that the distributions of the number of spikes in a burst per electrode (SBE) and burst duration (BD) contained one or two clusters of bursts (large and small). In cases where the second cluster was not obtained, the distribution had a decay or normal form. The distribution with two clusters had a bimodal form (Figure 3C). Depending on whether the activity contains large bursts or not, it was defined as type 1 or type 2 activity, respectively. The activity type was the same for both modules. The examples of two types of activity are shown in Figure 3D–G.

Two clusters of activity corresponded to super bursts, essentially constituting one unit of spontaneous activity. To study the dynamics and functional connections of type 1 activity, we considered only a cluster of large bursts. We found that the activity type of a modular network could change during development. In three networks, the activity type remained constant throughout development; in three networks, type 2 activity changed to type 1 on 25 DIV; and in one network, type 1 activity changed to type 2 on 25 DIV.

### 3.3. Burst Characteristics and Integrated Activity in Modular Network

**Figure 4:**
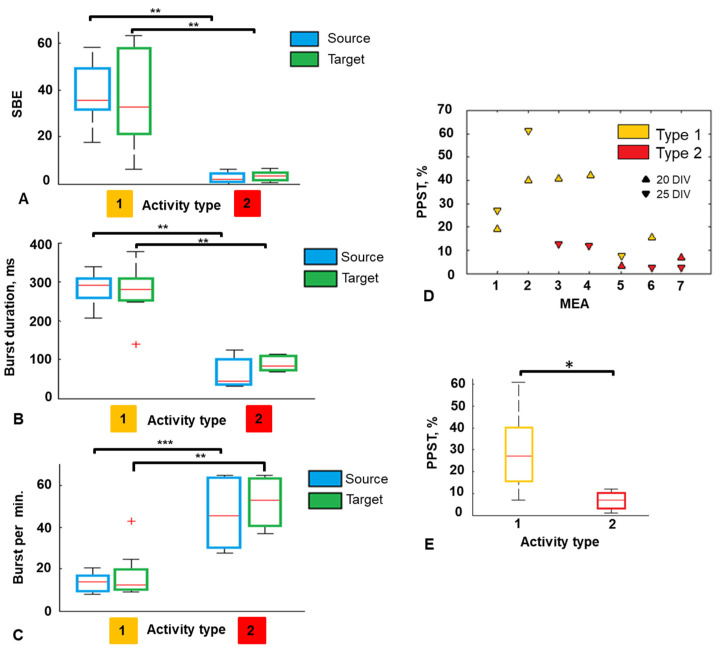
(**A**) SBE in the Source (blue) and the Target (green) modules estimated for two types of activity (n = 14, **—*p* < 0.001 Mann–Whitney test). (**B**) BD in the Source (blue) and the Target (green) modules estimated for two types of activity (n = 14, **—*p* < 0.001 Mann–Whitney test). (**C**) Number of bursts per minute in the Source (blue) and the Target (green) modules estimated for two types of activity (n = 14, **—*p* < 0.01, ***—*p* < 0.001 Mann–Whitney test). (**D**) Burst propagation probability from the Source to the Target module during development for 7 MEA. (**E**) Burst propagation probability from the Source to the Target module for the type 1 (yellow) and the type 2 (red) activity (n = 7 MEA, 14 rasters, *—*p* < 0.05 Mann–Whitney test). On each box, the central red line indicates the median, the ‘+’ symbol indicates the outliers.

The firing rate was not significantly different between the modules and the activity types. The median firing rate for type 1 activity was 114 spikes per sec in the Source module and 145 spikes per sec in the Target module, and it was 38 spikes per sec and 69 spikes per sec in the Source and the Target module, respectively, for type 2. The SBE of type 1 activity was significantly greater than in bursts of type 2 activity (*p* < 0.001 Mann–Whitney test). The median SBE of the Source module was 35 spikes for type 1 activity and 1.9 spikes for type 2 activity. Also, the SBE in the Target module was 32 spikes and 3.4 spikes for type 1 and type 2, respectively (Figure 4A).

Type 1 activity is characterized by significantly longer bursts than bursts of type 2 (*p* < 0.001 Mann–Whitney test). For type 1 activity, the median BD was 292 ms in the Source module and 280 ms in the Target module. For the type 2 activity, the BD was 43 ms in the Source and 82 ms in the Target module, respectively (Figure 4B). The number of bursts per minute was higher in networks with type 2 activity for both modules (Figure 4C).

We investigated how the intramodular bursting dynamics influenced the percentage of integration activity, i.e., the number of bursts propagated from the Source to the Target module (PPST). The PPST for type 1 activity was greater than 15% in most cases (seven out of eight) and less than 15% for type 2 activity (6 cases) (Figure 4D). The median value of PPST for type 1 activity was 27 (n = 8), while PPST for type 2 activity was significantly less, amounting to seven (n = 6; *p* < 0.05 Mann–Whitney test) (Figure 4E). The median values of the SBE and BD were higher in neural networks with type 1 activity by 9–18 times and 3–6 times, respectively. Type 1 activity is defined by the cluster of “large” bursts, which provide the PPTS 3.8 times greater than for type 2 activity.

**Figure 5:**
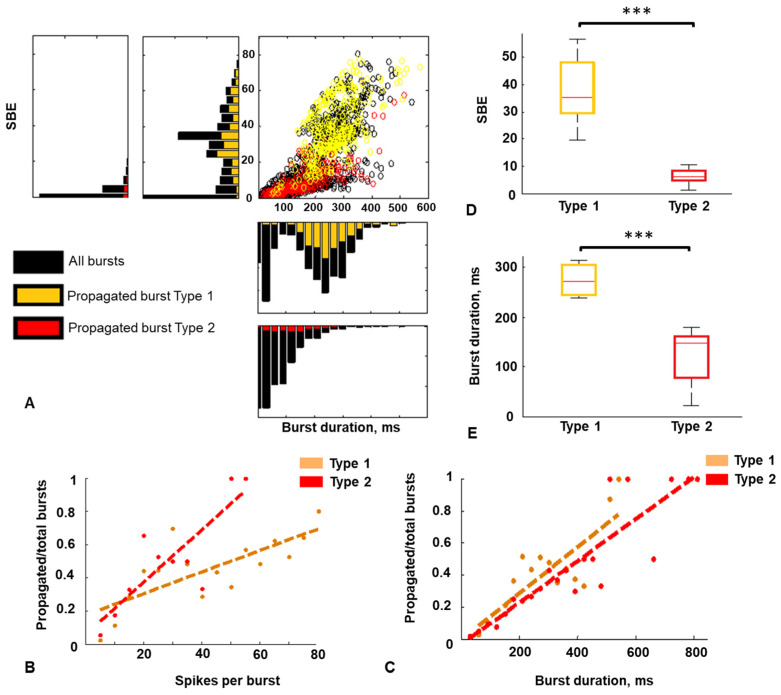
(**A**) Distribution of the SBE and BD in the Source chamber of type 1 and type 2 activity (bin equal to 5 spikes and 30 ms, respectively). The duration axis is presented in the range of 0–600 ms to visualize the shape of the distribution. (**B**) Ratio of SBE of propagated bursts to SBE of all bursts in the Source chamber and linear approximation. (**C**) Ratio of BD of propagated bursts to BD of all bursts in the Source chamber and linear approximation. (**D**) SBE of propagated bursts of two activity types (n = 14, ***—*p* < 0.001 Mann–Whitney test). (**E**) BD of propagated bursts of two activity types (n = 14, ***—*p* < 0.001 Mann–Whitney test).

Next, we investigated burst characteristics propagated from the Source to the Target module for both types of activity obtained in the Source module. We combined all activity recordings of the same type (1 or 2) and plot distributions of the SBE and the BD. The black distribution in Figure 5A corresponds to all bursts in recordings, the yellow to the propagated bursts in the type 1 activity, and the red to the propagated bursts in the type 2 activity. On the scatter plots, all bursts and propagated bursts are marked with circles and the same color coding. For both types, activity with a greater SBE and BD increased the probability of burst propagation (Figure 5B,C). The burst propagation probability monotonically depended on BD and spikes SBE.

The maximum of the SBE distribution was between 20 and 40, with a median value of 35 (Figure 5A,D). The BD of propagated bursts of type 1 activity had a pronounced maximum in the 230–300 ms band, with a median value of 271 ms (Figure 5A,E). Most of the propagated bursts belonged to the large burst cluster. As for neural networks with type 2 activity, the maximum values of the distribution of BD were between 30 and 160 ms (median value was 147 ms) (Figure 5A,D), and the maximum of SBE was near zero. The median value of the SBE and BD of the propagated bursts of type 1 activity was greater than in type 2 (*p* < 0.001, Mann–Whitney test) (Figure 5D,E).

### 3.4. Functional Connections in Networks with Two Activity

**Figure 6:**
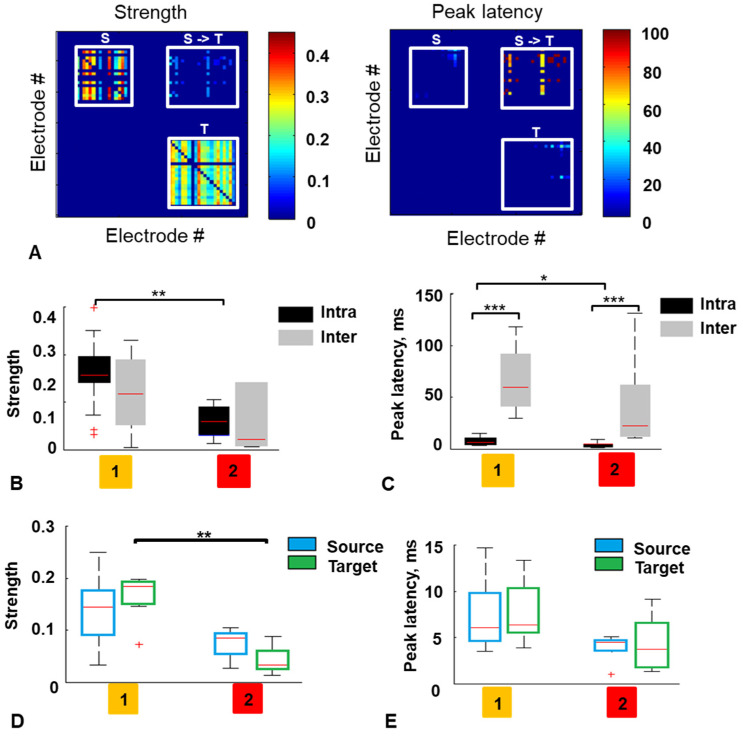
(**A**) Example of cross-correlation strength matrix (left) and peak latency matrix (right) for spontaneous spikes in the Source (S) and the Target (T) modules and for spikes propagated between modules (S->T). (**B**) Strength of connection into the modules and between modules (S->T) for two activity types. (**C**) Peak latency of spikes into the modules and between modules for two activity types. (**D**) The connection strength into the Source and the Target module for two types of activity. (**E**) The peak latency into the Source and the Target module for two types of activity. (n = 7 MEA, 21 rasters, ***—*p* < 0.001, **—*p* < 0.01, *—*p* < 0.05, Mann–Whitney test). On each box, the central red line indicates the median, the ‘+’ symbol indicates the outliers.

Then, we investigated whether the connections within and between the modules were different for the two types of activity using cross-correlation analysis [54]. The functional connections within modules and between them were evaluated by estimating the strength of neuronal connections and the peak latency of spiking activity (Figure 6A).

The comparison of intramodular connections showed that strength was higher for type 1 (0.15 and 0.05) and peak latency was longer for type 1 (6.1 ms and 4.4 ms, Figure 6B,C). The latency of the functional connections within the modules was less than between modules for both activity types (*p* < 0.001 Mann–Whitney test) (Figure 6C).

We did not find any difference comparing the Source and Target connectivity characteristics for each activity type. Such separate modular analysis showed the difference of the connection strength only in the Target module (median = 0.18 for type 1 and median = 0.03 for type 2; *p* < 0.01, Mann–Whitney test) (Figure 6D). There was no significant difference for the peak latency in both modules (Figure 6E).

It was shown that BD increases as a function of the complexity of the network circuitry [56]. The topology of the networks with type 1 activity is more complex and integrates more synaptic pathways in each module.

## 4. Discussion

We have created an in vitro neural network consisting of two spatially segregated modules connected in a directional manner (Figure 1). The activity of the Source module affects the activity of the Target module, but not vice versa. This network topology ensures the sequential processing of information, which is also observed in the brain, for example, in the laminar structure of the neocortex, the cerebro-cerebellar system, and the entorhinal-hippocampal circuit [57].

Directed connections allow us to assess the influence of the leading module on the functional structure of the whole network. Immunocytochemistry reveals a reduced number of inhibitory synapses in the Target network compared to the Source network (Figure 2). Unlike the Source, the Target network received input signals from another network with different activity dynamics during development through connections formed by the microchannels. It is known that synaptic development and transmission are impaired if external stimulation is blocked during in vivo development [58]. Conversely, optogenetic stimulation applied during the neurogenesis and synaptogenesis of motor neurons derived from embryonic stem cells in vitro led to increased neurite extension, clustering of synaptophysin, enhanced network synchronization, and improved evoked responses to stimulation within the network [59]. Previous studies have shown that spontaneous spiking activity and evoked responses increase in neuronal networks in vitro when exposed to external stimulation [60,61]. It has been suggested that synaptic scaling of excitatory and inhibitory receptors may differ in neuronal networks in vitro, depending on the presence or absence of external stimuli during development [60]. Our findings on the number of inhibitory synapses support this assumption. Moreover, our results are consistent with in vivo experiments in which visual deprivation in rats did not alter excitatory cortical connections but enhanced inhibitory feedback [62]. However, unlike chronic electrical stimulation [60,61], inputs from a different subnetwork did not lead to changes in the level of spontaneous activity in our experiments. The lack of differences in the activity levels between the Source and Target networks can be explained by homeostatic regulation mechanisms [58,63]. The dominance of network burst propagation was independent of the balance of firing rates between two bidirectionally connected subnetworks [46], which is consistent with our observations. Furthermore, our results align with previous findings showing a decrease in inhibitory synapses on distal dendrites in local neural networks developing under conditions of chemically induced hyperactivity [64]. Presumably, the mechanism behind this phenomenon could be related to the specific location of synapses, such as a decrease in the number of inhibitory synapses on dendrites and a simultaneous increase in somas [64]. Our results confirm structural rearrangements in neural networks influenced by external activity, promoting optimal interaction between Source and Target modules.

In brain neural networks, the balance between local population subgroup activity and spreading activity between subgroups is crucial for cognitive functions [20,65,66]. Simple motor tasks exhibit more segregated activity, while complex cognitive tasks involving memorization and attention maintenance show more integrated activity [67].

In our in vitro neural networks, we observed a combination of integrated and segregated activity, expressed through spontaneous bursts involving a single module or propagating from the Source to the Target module. The directionality of burst propagation was confirmed by estimating the percentage of bursts propagated (PP) forward and backward relative to the total number of bursts in the module. Almost all integrated bursts propagated in the Source–Target direction, but not vice versa. Similar results were obtained in our previous study [55].

Numerous in vitro studies have shown that integration activity increases with the number of connections between modules (subnetworks), regulated by the number and geometry of channels connecting the chambers of microfluidic chips [31,40,41,42,43], but there is almost no research on the interplay of intramodular network activity and intermodular functional organization. In our modular networks, the identity of cultures was ensured by the same chamber size, number of microchannels, and cell density. Earlier, S. Potter showed that neural cultures exhibit different spontaneous bursts under the same initial conditions [68]. In our networks, spontaneous activity varied between networks on different DIV and led to different levels of interaction between modules (PPST) (Figure 4E). We hypothesized that the character of spontaneous activity can lead to different intermodular interactions.

We identified two types of spontaneous bursting activity based on burst characteristics, such as the number of spikes in a burst and burst duration (Figure 4). Integrated activity was more represented in networks with type 1 activity. The integrated bursts of type 1 activity were longer and contained more spikes than type 2 (Figure 5). However, the burst propagation probability monotonically increased with burst duration and spikes per burst. Similar results were obtained in a neural network of 60 modules, where bursts activating the neighboring module had a higher intensity, expressed as the amplitude of spikes in a burst [32].

We further demonstrated that functional structure was different for networks with type 1 and type 2 activity. For each type, differences in time delays within and between modules inherent in modular networks were observed, ensuring the separation of intra- and inter-modular information processing. The lower delay within modules for both types of activity is associated with a greater number and density of connections compared to intermodular connections, physically limited by the number and size of microchannels. Networks with type 1 activity had stronger intramodular connections and larger peak latency within modules, possibly reflecting the presence of many polysynaptic connections (Figure 6). Interestingly, the difference in the strength of intra- and inter-modular connections was found only for type 1. In type 2, the connection strength within modules was so low that it did not differ from the strength of intermodular connections. However, the strength and peak delay of intermodular connections were similar for the two types.

We showed that in vitro modular networks can reproduce different modes of activity without manipulating the number of connections between modules. The two types of activity of the Source module essentially correspond to two dynamic modes of operation of the modular network: more and less synchronous. A network can persist in one mode during maturation or can transition between modes (Figure 4E). A change in the type of activity can occur due to the maturation of synaptic connections or their elongation, reconfiguration of synaptic weights, and the level of excitability of neurons.

The type of activity is also reflected in intramodular connectivity, type 1 characterized by stronger connections at long polysynaptic delays, and type 2 resulting from weak connections at short delays, leading to a more clustered and fragmented architecture.

The level of integrated activity in hierarchically connected modular networks depends on the strength of functional connectivity within the modules. Stronger intramodular connectivity leads to the emergence of large bursts in the Source module, spreading along intermodular connections and involving the Target module in the overall activity. We expect that modular networks with a high level of integral activity will more effectively reproduce such simple functions as selectivity of response to a stimulus at different spatial points of the Source module [30].

Neuroengineering methods recapitulate the topological organization and hierarchical connections of neural circuits similar to brain networks, which are not available for direct investigation. Our hierarchically modular platform can be used to study function-structure relationships and efficient information processing in neural networks. Scaling our approach into several connected modules will allow the study of the involvement of neurons in different layers of information processing. Combining our platform with iPSC culture technologies will make it useful for pharmacological and neurodegenerative disease research.

## Data Availability

The data presented in this study are available on request from the corresponding author. The code used to produce the results and experimental data of spiking activity are available in a public GitHub repository at https://github.com/alex-pimashkin/MeaData (accessed on 25 May 2024).
